# Practical PCR genotyping protocols for *Plasmodium vivax *using *Pvcs *and *Pvmsp1*

**DOI:** 10.1186/1475-2875-4-20

**Published:** 2005-04-27

**Authors:** Mallika Imwong, Sasithon Pukrittayakamee, Anne Charlotte Grüner, Laurent Rénia, Frank Letourneur, Sornchai Looareesuwan, Nicholas J White, Georges Snounou

**Affiliations:** 1 Department of Clinical Tropical medicine, Faculty of Tropical Medicine, Mahidol University, Bangkok, Thailand; 2 Département d'Immunologie, INSERM U567, CNRS UMR8104, Institut Cochin, Université René Descartes, Paris 75014, France; 3 Laboratoire Commun de Séquençage, Institut Cochin, Université René Descartes, Paris 75014, France; 4 Wellcome Unit, Faculty of Tropical Medicine, Mahidol University, Bangkok, Thailand; 5 Centre for Vaccinology and Tropical Medicine, Churchill Hospital, Oxford, UK; 6 Unité de Parasitologie Bio-Médicale, CNRS URA2851, Institut Pasteur, Paris, France; 7 Parasitologie Comparée et Modèles Expérimentaux USM307, CNRS IFR101, Muséum National d'Histoire Naturelle, CP52, 61 Rue Buffon, 75231 Paris Cedex 05, Paris, France

## Abstract

**Background:**

*Plasmodium vivax *is the second most prevalent malaria parasite affecting more than 75 million people each year, mostly in South America and Asia. In addition to major morbidity this parasite is associated with relapses and a reduction in birthweight. The emergence and spread of drug resistance in *Plasmodium falciparum *is a major factor in the resurgence of this parasite. *P. vivax *resistance to drugs has more recently emerged and monitoring the situation would be helped, as for *P. falciparum*, by molecular methods that can be used to characterize parasites in field studies and drug efficacy trials.

**Methods:**

Practical PCR genotyping protocols based on polymorphic loci present in two *P. vivax *genetic markers, *Pvcs *and *Pvmsp1*, were developed. The methodology was evaluated using 100 *P. vivax *isolates collected in Thailand.

**Results and Discussion:**

Analysis revealed that *P. vivax *populations in Thailand are highly diverse genetically, with mixed genotype infections found in 26 % of the samples (average multiplicity of infection = 1.29). A large number of distinguishable alleles were found for the two markers, 23 for *Pvcs *and 36 for *Pvmsp1*. These were generally randomly distributed amongst the isolates. A total of 68 distinct genotypes could be enumerated in the 74 isolates with a multiplicity of infection of 1.

**Conclusion:**

These results indicate that the genotyping protocols presented can be useful in the assessment of *in vivo *drug efficacy clinical trials conducted in endemic areas and for epidemiological studies of *P. vivax *infections.

## Background

*Plasmodium vivax *is globally distributed and is the dominant species in many countries. Infection by this species is generally regarded as benign and mortality is a rare outcome. 1.2 billion inhabitants of the South East Asian and Pacific countries are at risk from malaria transmission, representing about a third of world population exposed to *Plasmodium *parasites [1,2]. Most of the populations at risk in these countries (corresponding to the WHO regional groupings SEARO and WPRO) reside in hypoendemic and mesoendemic areas, where half of the recorded infections are due to *P. vivax*, suggesting that the global burden of vivax malaria morbidity must be close to that of falciparum malaria. It has recently been established that *P. vivax *infections during pregnancy are associated with reduced birthweight [3], and thereby, increase the risk of neonatal deaths. Thus, the urgency to develop and implement measures to control *P. vivax *should equal that attending *P. falciparum*.

The emergence and global spread of *P. falciparum *parasites resistant to chloroquine and pyrimethamine – sulfadoxine has led to an increase in morbidity and mortality [4], forcing many countries to abandon these relatively cheap drugs. Recent reports of *P. vivax *resistance to these drugs [5-13] are of concern and steps to restrict the emergence of drug resistance need to be taken. A critical component of malaria control is surveillance for resistance. Despite some disadvantages, *in vitro *assessment of the susceptibility of *P. falciparum *to antimalarial drugs provides a rapid means to monitor general levels of drug resistance. Although protocols to culture *P. vivax *parasites *ex-vivo *have been developed [14,15], they are still unsuited for routine assays. Thus, well conducted clinical trials, an essential component of resistance monitoring, remain the only alternative for *P. vivax*. However, *in vivo *drug efficacy studies conducted in endemic areas are to some extent compromised by their inability to distinguish true recrudescences, i.e. treatment failures, from re-infections which become clinically or parasitologically manifest during the follow-up period, i.e. treatment successes. This has been largely overcome for *P. falciparum *by the application of genotyping based on well characterized polymorphic regions within the gene encoding *msp1*, *msp2 *and *glurp *[16].

A similar approach has not been adopted for *P. vivax*, a parasite species less well-studied at the molecular level than *P. falciparum *[17]. Four polymorphic *P. vivax *single copy genes have been independently used for molecular epidemiological studies: *Pvgam1 *coding for a gametocyte antigen, *Pvcs *coding for the circumsporozoite protein, *Pvmsp1 *and *Pvmsp3α *coding for the merozoite surface proteins 1 and 3 alpha, respectively. Amplification of *Pvgam1 *was found to be associated with artefacts [18] and should thus be excluded as a genetic marker. The suitability of *Pvmsp3α *as a marker has been recently validated [19]. The extent of diversity found for the remaining two genes was mainly established through sequencing of amplified fragments.

The objective of this work was to develop field applicable methods of genotyping which could also complement clinical trials in vivax malaria. The highly polymorphic genes *Pvcs *and *Pvmsp1 *were therefore selected to develop protocols for polymerase chain reaction (PCR) amplification and subsequent analysis of the polymorphic regions. The methodology was then assessed and validated with samples obtained from patients who acquired a *P. vivax *infection in Thailand.

## Materials and Methods

### Blood samples

Blood samples were collected from adult patients with symptomatic *P. vivax *malaria admitted to the Bangkok Hospital for Tropical Diseases, Thailand between 1995 to 1998 (n = 100). All patients gave fully informed consent to enrolment in these studies which were approved by the Ethics committee of the Faculty of Tropical Medicine, Mahidol university. Diagnosis was established by microscopy at the malaria laboratory through examination of thin and thick blood smears, stained with Field's stain. The blood samples were kept at -30°C until DNA extraction.

### DNA template preparation

DNA was purified from blood sample using the commercially available DNA Blood Kit (QIAGEN, Germany). The final volume of the DNA solution used as a template for the amplification reactions was such that 1 μL corresponded to 5 μL of whole blood. Confirmation of the microscopic diagnosis as *P. vivax *and testing for the presence of other *Plasmodium *species were achieved by a previously described PCR-based protocol [20].

### Amplification protocols

In order to increase the sensitivity of amplification, a nested or semi-nested PCR approach was adopted for all the fragments amplified except for one of the three *Pvmsp1 *regions. Oligonucleotide primers were designed using published sequences of *Pvcs *and of *Pvmsp1 *(their sequences are presented in Table 1). The optimal Mg2+ concentrations, annealing temperatures and numbers of cycles were individually determined for the different primer pairs and are presented in Table 1.

**Table 1:** Primers used for genotyping *P. vivax *parasites.

**Gene**	**Primer**	**Sequence^a^**	**mM Mg^2+ b^**		**Annealing °C ^b^**		**Cycles^b^**	
			**1^st^**	**2^nd^**	**1^st^**	**2^nd^**	**1^st^**	**2^nd^**

								
***Pvcs***	VCS-OF	ATGTAGATCTGTCCAAGGCCATAAA	1		58		25	
	VCS-OR	TAATTGAATAATGCTAGGACTAACAATATG	1		58		25	
	VCS-NF	GCAGAACCAAAAAATCCACGTGAAAATAAG		1		62		30
	VCS-NR	CCAACGGTAGCTCTAACTTTATCTAGGTAT		1		62		30
								
***Pvmsp1 *F1**	VM1-O1R	CCACTCCATGAAACTGAAGTGTTTA	2		68		25	
	VM1-N1F	CGATATTGGAAAATTGGAGACCTTCATCAC	2	2	68	68	25	30
	VM1-N1R	CTTTTGCGCCTCCTCCAGCTGGCTCGTGT		2		68		30
								
***Pvmsp1 *F2**	VM1-O2F	GATGGAAAGCAACCGAAGAAGGGAAT	1		50		25	
	VM1-O2R	AGCTTGTACTTTCCATAGTGGTCCAG	1	1	50	64	25	35
	VM1-N2F	AAAATCGAGAGCATGATCGCCACTGAGAAG		1		64		35
								
***Pvmsp1 *F3**	VM1-3F	CAAGCCTACCAAGAATTGATCCCCAA	1		66		42	
	VM1-3R	ATTACTTTGTCGTAGTCCTCGGCGTAGTCC	1		66		42	

^a ^Sequences are provided 5'- to 3'-end.p

^b ^The two columns indicate conditions for the primary and secondary amplification reactions.

All amplification reactions were carried out in a total volume of 20 μL and in the presence of 10 mM Tris-HCl, pH 8.3, 50 mM KCl, 250 nM of each oligonucleotide primers, 125 μM of each of the four dNTPs, and 0.4 units of AmpliTaq polymerase (Perkin Elmer Cetus, USA). Primary amplification reactions were initiated with one μL of the template genomic DNA prepared from the blood samples, and the one μL of the product of these reactions was used to initiate the secondary amplification reactions. The cycling parameters for PCR were as follows: an initial denaturation step at 95°C for five minutes preceded the cycles of annealing at a temperature defined for each primer pair (Table 1) for two minutes, extension at 72°C for two minutes, and denaturation at 94°C for one minute. After a final annealing step followed by five minutes of extension, the reaction was stopped. PCR products were stored at 4°C until analysis.

The lack of cross-contamination was monitored by the inclusion of multiple, randomly distributed, negative control samples (human DNA or no template) in each amplification run. A subset of the samples were analysed in triplicate in order to confirm the consistency of the results obtained.

### Analysis of the amplification product

The DNA fragments obtained following PCR were analysed by electrophoresis in agarose gels. For direct analysis of the fragments, 10 μL of the amplified PCR product were mixed with two μL of loading buffer and applied to 2 % agarose gels. For restriction fragment length polymorphism analysis of the PCR products (PCR-RFLP), 10 μL of the amplified PCR product were first digested with a restriction enzyme (New England Biolabs Inc., UK) according to the suppliers specifications, for three hours in a total volume of 20 μL, before adding five μL of loading buffer and applying to 1.5 % or 1.8 % agarose gels. Electrophoresis was performed in TBE buffer, and the DNA was visualised on an ultraviolet transilluminator following ethidium bromide staining. The size of the amplified fragments was estimated by comparison to a 100 bp ladder marker set.

Selected amplified fragments were purified using QIAquick gel extraction kits (QIAGEN, Germany) and cloned using the TOPO -TA Cloning kit (Invitrogen, U.S.A.). Plasmid DNA containing the fragment was purified from positive bacterial colonies using the QIAquick Miniprep spin kit (QIAGEN, Germany) and sequenced using an ABI automated sequencer. Sequence alignments were performed using the Gene Jockey II program (Biosoft, United Kingdom).

## Results

### Specificity and sensitivity of the amplification reactions

Primers specific to *Pvcs *and *Pvmsp1 *were designed to hybridize to sequences conserved in all variants known at that time. The specificity of all the primer pairs was confirmed since amplification products were only observed when *P. vivax *DNA was included in the reaction, but not when genomic DNA from *P. falciparum*, *Plasmodium malariae*, *Plasmodium ovale *or humans were used as a template. A set of a 10-fold serial dilution of a template prepared from a blood sample containing *P. vivax *only, calibrated using a previously described protocol [20], was used to assess the sensitivity of the reactions. Consistent detection of 10 parasite genomes was achieved for *Pvcs*-specific primers, and of 50 parasite genomes for *Pvmsp1*-specific primers. These levels of sensitivity were in agreement with subsequent analyses of samples harbouring a known number of parasites.

### Genotyping using *Pvcs*

**Figure 1:**
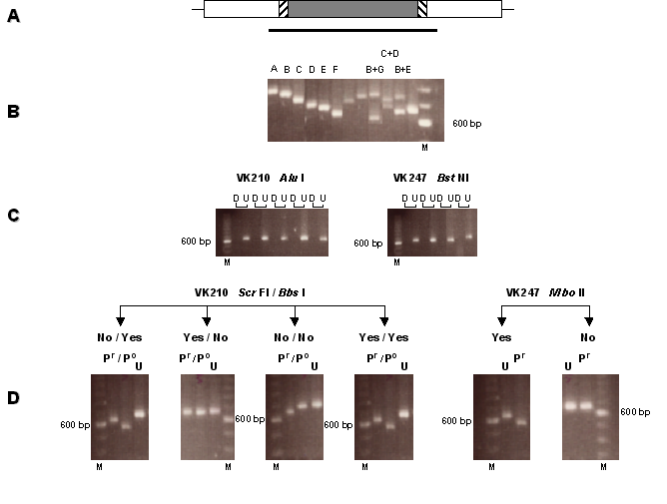
A. Schematic representation of the *Pvcs *gene, which consists of a conserved region (blank box) flanking a repeated region (grey box) which is itself flanked by pre- and post-repeat specific sequences (cross-hatched boxes). The bold horizontal line represents the PCR-amplified fragment used for further analysis. B. Example of the fragments (denoted A to G) of distinct size observed in different isolates, as single or as mixed infections (some lanes were not labelled because of lack of space). C. Fragments digested (D) or undigested (U) with *Alu *I or *BstN *I, two restriction enzymes with multiples sites in VK 210 type or VK247 type repeat sequences, respectively. Digestion cuts the fragment in small fragments of less than 150 bp in size. D. PCR-RFLP using specific restriction enzymes analysis for the presence/absence (yes/no) of specific pre- and post-repeat sequences in fragments carrying the VK210 or VK247 type repeats. Undigested fragments are denoted by (U) and P^r ^and P^o ^denote digests to determine the presence of pre- and post-repeat sequence types, respectively. A 100 bp ladder, where the 600 bp band stains most intensely, was used a molecular weight marker (M) for all the gels.

The circumsporozoite protein gene of *P. vivax*, as for other *Plasmodium *species, comprises a central repetitive domain flanked by two conserved domains. The majority of variations observed in *Pvcs *occur in the repeat region and the immediate pre- and post-repeat sequences. Thus the genotyping strategy was focused on an amplified fragment spanning these regions (Fig. 1A). In a given parasite line, the repetitive domain is composed of a 27 bp element repeated a variable number of times. Variations in the number of repeated elements result in size polymorphisms amenable to detection by electrophoresis. Seven allelic types distinguishable by size were observed in the Thai *P. vivax *isolates analysed (Fig. 1B), a number consistent with the variations in the number of repeat elements (15 to 21) previously observed for *Pvcs *[21,22]. In *P. vivax*, two types of repeat elements are found, VK210 and VK247 [23], and a given *Pvcs *gene will exclusively bear either the VK210 type (type I repeats based on GDRADGQPA) or the VK247 type (type II repeats based on ANGAGNQPG). In order to differentiate between amplified fragments carrying the VK210 from those carrying the VK247 repeat element, the exclusive presence of *Alu *I recognition sites in the VK210-type and of *Bst *NI recognition sites in the VK247-type repeated sequences were exploited. Thus, following digestion with the appropriate enzyme, the amplified fragments are degraded into fragments of < 150 bp, easily distinguishable from the original fragments of > 700 bp (Fig. 1C). In this manner the PCR fragments obtained from an isolate can be grouped by size and by repeat type.

In order to increase the genotyping resolution of *Pvcs *as a marker, so as to subdivide the *P. vivax *population into a larger number of *Pvcs *allelic types, sequence variations previously observed in the pre- and post-repeat regions [24] were used to develop an additional PCR-RFLP protocol (Fig. 1D). For some *Pvcs *genes, the pre-repeat consists of the Region I sequence (KLKQP) that directly abuts the repeated region, while for others amino acid residues are inserted between the two regions, namely a T, V or A residue for VK210-type *Pvcs *genes, or ED residues for VK247-type *Pvcs *genes. The *Scr *FI restriction endonuclease cuts VK210-type *Pvcs *fragments that do not have the T/A/V insertion following Region I, thus shortening them by 41 bp. VK247-type *Pvcs *bearing the ED insertion after Region I are cut by the *Mbo *II restriction endonuclease and the amplified fragment is thereby shortened by 55 bp. For the post-repeat region, a 36 bp insertion has been found for some VK210-type *Pvcs*. This insertion harbours a recognition site for the *Bbs *I enzyme, and digestion truncates the corresponding PCR fragments by 115 bp.

**Figure 2:**
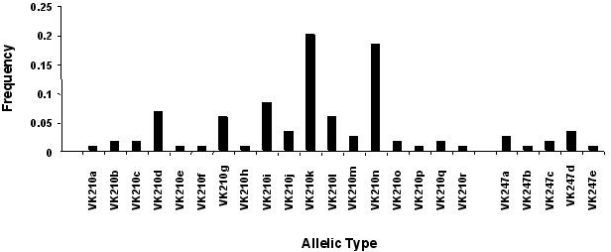
Allele frequency of the distinct allelic variants of *Pvcs *observed in the 100 isolates from Thailand. Allelic types were defined according to repeat type (VK210 or VK247), fragment size and the presence or absence of defined pre- and post-repeat sequences.

Genotyping of the 100 *P. vivax *isolates collected from Thailand was achieved successfully using the protocols described above. In total, parasites with a *Pvcs *bearing the VK210 type repeats only were found in 90 isolates (representing seven size polymorphisms). Parasites with a *Pvcs *bearing the VK247-type repeats only were found in nine isolates (representing only three size polymorphisms), and in one isolate parasites of both types were found. Thus, 10 different allelic forms of *Pvcs *were detected by simple analyses of the fragment size and repeat type. When associated with RFLP analysis of the pre- and post-repeat types, these could be divided into 23 different allelic types (Table 2), 18 for the VK210 type and five for the VK247 type. Mixed genotype infections were found in 20 of the isolates, for each of which the multiplicity of infection (MOI), i.e. the total number of different allelic types observed, was two. Thus, for the samples analysed, a total of 120 bands were observed, with an mean MOI of 1.2. The allelic variants were generally randomly distributed between the 120 bands; the highest frequencies, 0.2 and 0.18, were found for VK210k and VK210n, respectively (Fig 2).

**Table 2:** Frequency of *Pvcs *allelic variants classed by size, repeat type and presence of pre- and post-repeat insertions.

**Allele**	**Size^a^**	**Pre-repeat^b^**	**Post-repeat^b^**	**n**	**Frequency^c^**
**VK210a**	A	Yes	No	1	**0.008**
**VK210b**	B	Yes	No	8	**0.067**
**VK210c**	C	Yes	No	7	**0.058**
**VK210d**	C	No	No	1	**0.008**
**VK210e**	C	Yes	Yes	1	**0.008**
**VK210f**	D	Yes	No	24	**0.200**
**VK210g**	D	No	No	10	**0.083**
**VK210h**	D	No	Yes	4	**0.033**
**VK210i**	E	Yes	No	22	**0.183**
**VK210j**	E	No	No	7	**0.058**
**VK210k**	E	No	Yes	3	**0.025**
**VK210l**	F	No	No	2	**0.017**
**VK210m**	F	No	Yes	1	**0.008**
**VK210n**	F	Yes	No	2	**0.017**
**VK210o**	B	No	No	2	**0.017**
**VK210p**	B	No	Yes	2	**0.017**
**VK210q**	G	No	No	1	**0.008**
**VK210r**	C	No	Yes	1	**0.008**
					
**VK247a**	B	In	ND	3	**0.025**
**VK247b**	C	In	ND	2	**0.017**
**VK247c**	D	In	ND	4	**0.033**
**VK247d**	B	No	ND	1	**0.008**
**VK247e**	E	No	ND	1	**0.008**

^**a **^The fragments were assigned to different size bins (labelled A to G in decreasing order) by visual inspection.

^**b **^"Yes" and "No" respectively denote the presence or absence of insertions in the pre- and post-repeat regions as ascertained by PCR-RFLP analysis.

^**c **^Calculated for the 120 bands observed in the 100 Thai isolates analysed.

### Genotyping using *Pvmsp1*

**Figure 3:**
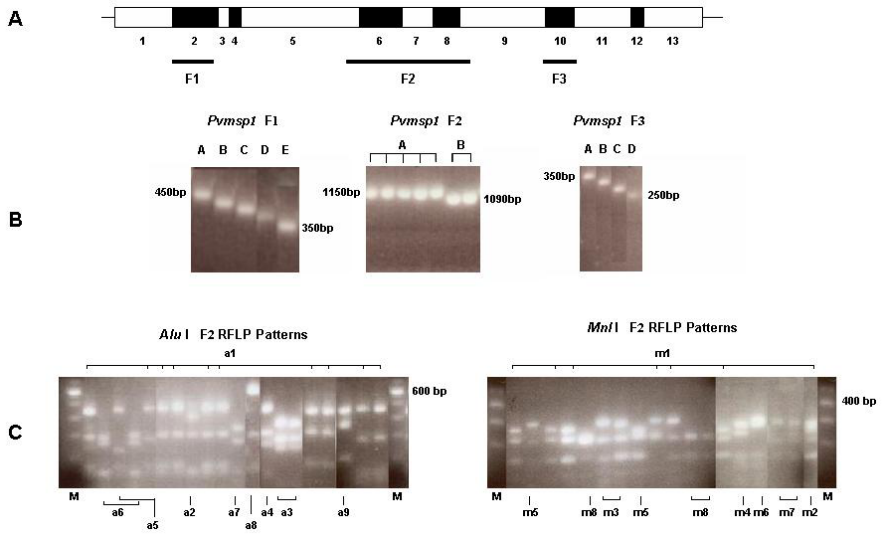
A. Schematic representation of the *Pvmsp1 *gene for the localization of interallele conserved blocks (blank boxes) and variable blocks (black boxes). The position of the three amplified segments (F1, F2 and F3) is indicated by the horizontal bold lines. B. Gel electrophoresis of PCR products of a selection of fragments, corresponding to the three segments, amplified from different isolates. The molecular size of the largest and smallest bands are indicated. C. Analysis of the diversity of the F2 segment by PCR-RFLP using *Alu *I or *Mnl *I. A selection of lanes is labelled to indicate the type of variant observed (corresponding to the nomenclature in Table 3). A 100 bp ladder was used a molecular weight marker (M) for all the gels.

The *Pvmsp1 *gene encodes a polypeptide of about 1,720 amino acids [25,26], and sequence comparison revealed 13 regions of interallele conserved blocks and variable blocks [27]. Three main regions of sequence divergence were found through comparison of the full length *Pvmsp1 *sequences from two distinct *P. vivax *lines (Sal-1 and Belem). Three segments (labelled F1 to F3) corresponding to these regions were thus amplified for further analysis (Fig. 3A).

For the 100 Thai *P. vivax *isolates, five distinguishable size variants were observed for F1 (ca. 350 bp – 450 bp), and four for F3 (ca. 250 bp – 350 bp), but only two were observed for the larger (ca. 1087 bp -1150 kb) F2 segment (Fig. 3B). One allelic variant dominated in frequency for each of the three amplified segments (Table 3): band C for F1 (42 % of the bands observed), band A for F2 (78 %) and band D for F3 (76 %). Mixed genotype infections were observed infrequently and only for the F1 (n = 3, 2 MOI of 2 and 1 MOI of 3) and for the F3 (n = 6, 4 MOI of 2 and 2 MOI of 3) segments.

**Table 3:** Frequency of *Pvmsp1 *allelic variants found in segments F1, F2 and F3.

**F1**			**F2**			**F3**		
**Variant**	**n**	**Frequency**	**Variant^a^**	**n**	**Frequency**	**Variant**	**n**	**Frequency**

								
**A**	4	**0.038**	**Aa1**	50	**0.5**	**A**	1	**0.009**
**B**	19	**0.183**	**Aa3**	6	**0.06**	**B**	12	**0.112**
**C**	44	**0.423**	**Aa6**	15	**0.15**	**C**	12	**0.112**
**D**	22	**0.212**	**Aa7**	4	**0.04**	**D**	82	**0.766**
**E**	15	**0.144**	**Aa8**	1	**0.01**			
			**Aa9**	1	**0.01**			
			**Aa10**	1	**0.01**			
			**Ba1**	6	**0.06**			
			**Ba2**	1	**0.01**			
			**Ba3**	5	**0.05**			
			**Ba5**	2	**0.02**			
			**Ba6**	4	**0.04**			
			**Ba7**	3	**0.03**			
			**Ba10**	1	**0.01**			
								
			**Am1**	38	**0.38**			
			**Am2**	6	**0.06**			
			**Am3**	2	**0.02**			
			**Am4**	4	**0.04**			
			**Am5**	11	**0.11**			
			**Am6**	2	**0.02**			
			**Am8**	14	**0.14**			
			**Am9**	1	**0.01**			
			**Bm1**	6	**0.06**			
			**Bm2**	3	**0.03**			
			**Bm3**	7	**0.07**			
			**Bm4**	2	**0.02**			
			**Bm5**	3	**0.03**			
			**Bm8**	1	**0.01**			

^a ^RFLP patterns obtained by digestion with *Alu *I or *Mn*l I were numbered in two series a1, a2, etc.. and m1, m2 etc..., respectively. A given pattern could be obtained irrespective of the size of the fragment amplified (A or B).

**Figure 4:**
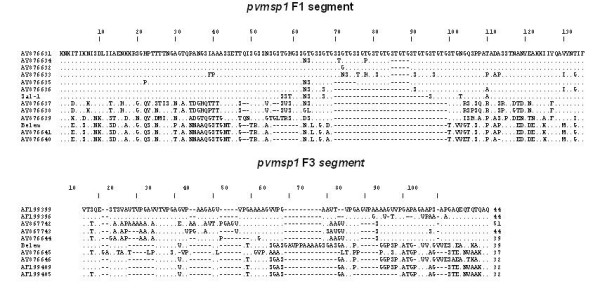
Alignment of amino acid sequences of amplified F1 and F3 fragments of *Pvmsp1*. Sequences were aligned against the largest fragment obtained; dots represent identical residues and dashes represent gaps. Sequences obtained during this study are labelled by their GenBank accession numbers, while the Belem and Sal-1 sequences had been previously published (AF435594 and AF435593, respectively).

A selection of amplified product from F1 (n = 18) and F3 (n = 8) representative of all the size variants were sequenced and compared to previously published sequences (Fig. 4). The number of distinct F1 variants was found to be higher than that revealed by electrophoretic separation, since 11 different allelic variants were observed for the 18 fragments derived from the Thai isolates. Sequence differences were observed within the variants assigned to the B, D or E size classes. Some of the bands within each class might be distinguished by the use of higher resolution agarose gels, though others had the same size and only exhibited subtle sequence differences. A similar pattern was observed for the F3 fragments, where sequencing distinguished six different variants.

**Figure 5:**
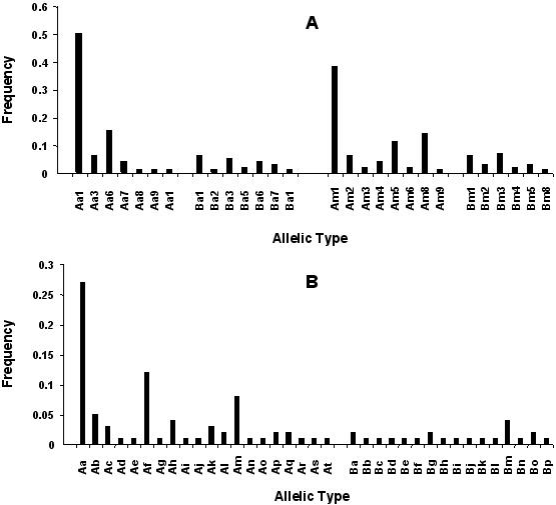
Allele frequency of the distinct allelic variants of the F2 segment of *Pvmsp1 *observed in the 100 isolates from Thailand. Allelic variants were defined according to the RFLP patterns observed. A. Variants were divided according to the digestions patterns obtained individually with each of the two restriction enzymes *Alu *I (a1, a2, etc...) or *Mnl *I (m1, m2, etc...) for A or B, the two different sized fragments amplified, as in Table 3. B) Variants were classed according to size and the combined RFLP patterns obtained for both restriction enzymes (*Alu *I and *Mnl *I), as in Table 4.

The F2 segment, which was comparatively poorly polymorphic in size in the Thai isolates, encompassed regions established as polymorphic in previous studies [27]. An RFLP strategy was thus adopted to distinguish between the different allelic variants. The restriction endonucleases *Alu *I and *Mnl *I which recognize multiple sites in F2, were used to reveal extensive polymorphism at the nucleotide level (Fig. 3C). Thus, nine different *Alu *I and eight different *Mnl *I RFLP patterns were observed in the 100 Thai isolates (Table 3). The occurrence of patterns indicative of partial digestion, or the presence of mixed F2 genotypes in individual samples were excluded since the sum of the RFLP fragments' sizes was not found to be greater than that of the uncut product for any isolate and the patterns observed were not unique to isolates where mixed genotypes were detected through size polymorphism of the F1 or F3 segments. When the data from both analyses (size and RFLP) were combined, 36 *Pvmsp1 *F2 allelic variants could be differentiated (Table 4). When the frequency of the allelic variants was considered individually for the two RFLP patterns, 50 % and 38 % of the isolates were of a single *Alu *I- and *Mnl *I-classified allelic variant, respectively. The remaining isolates were randomly distributed between the different variants (Fig. 5A). However, by combining the results for both restriction enzymes, the most dominant allele was found in only 27 % of the isolates (Fig. 5B), while the remainder were found at lower frequency (5 % or less).

**Table 4:** Frequency of the F2 *Pvmsp1 *allelic variants classed following RFLP analysis.

**Variant**	**Size**	***Alu *I Pattern number**	***Mnl *Pattern number**	**n^a^**	**Frequency**
**Aa**	A	1	1	27	**0.27**
					
**Ab**	A	1	2	5	**0.05**
**Ac**	A	1	4	3	**0.03**
**Ad**	A	1	5	1	**0.01**
**Ae**	A	1	6	1	**0.01**
**Af**	A	1	8	12	**0.12**
**Ag**	A	1	9	1	**0.01**
**Ah**	A	3	1	4	**0.04**
**Ai**	A	3	2	1	**0.01**
**Aj**	A	3	4	1	**0.01**
**Ak**	A	6	1	3	**0.03**
**Al**	A	6	3	2	**0.02**
**Am**	A	6	5	8	**0.08**
**An**	A	6	6	1	**0.01**
**Ao**	A	6	8	1	**0.01**
**Ap**	A	7	1	2	**0.02**
**Aq**	A	7	5	2	**0.02**
**Ar**	A	8	1	1	**0.01**
**As**	A	9	1	1	**0.01**
**At**	A	10	8	1	**0.01**
					
**Ba**	B	1	1	2	**0.02**
**Bb**	B	1	2	1	**0.01**
**Bc**	B	1	3	1	**0.01**
**Bd**	B	1	4	1	**0.01**
**Be**	B	1	8	1	**0.01**
**Bf**	B	2	1	1	**0.01**
**Bg**	B	3	1	2	**0.02**
**Bh**	B	3	2	1	**0.01**
**Bi**	B	3	3	1	**0.01**
**Bj**	B	3	4	1	**0.01**
**Bk**	B	5	1	1	**0.01**
**Bl**	B	5	2	1	**0.01**
**Bm**	B	6	3	4	**0.04**
**Bn**	B	7	3	1	**0.01**
**Bo**	B	7	5	2	**0.02**
**Bp**	B	10	5	1	**0.01**

^**a **^Number of isolates where a particular pattern was observed amongst the 100 Thai isolates analysed

### Two locus genotyping

When the genotype analyses from the four polymorphic regions of the two genes were combined, the 100 isolates collected from Thai patients proved to be highly genetically diverse. Mixed genotype infections were detected in 26 of the isolates, mainly through the *Pvcs *gene (n = 20). Only one of the isolates identified as mixed genotype infections by *Pvmsp1 *(n = 7) was classed as such by *Pvcs *analysis. The mean MOI for isolates with a mixed genotype was 2.11, and that for the complete set of isolates was 1.29.

There were 74 isolates where a single genotype was detected after full analysis of the *Pvcs *and *Pvmsp1 *markers. Comparison of the genotyping patterns obtained for these isolates allows to ascertain the relative contribution of the polymorphic loci to distinguish between *P. vivax *populations. When the complete genotyping analyses are taken into account, a total of 68 distinct genotypes are enumerated. Sixty-three genotypes were observed in only one isolate, with a maximum of three isolates sharing the same genotype (highest genotype frequency = 0.040). No evidence for linkage disequilibrium could be detected when *Pvcs *and *Pvmsp1 *were considered. If the results from the *Pvmsp1 *F1 segment are omitted from the analysis, 58 distinct genotypes would be found, with a maximum of four isolates sharing the same genotype (highest genotype frequency = 0.054). Incremental omission of the *Pvmsp1 *F3 segment followed by that of the *Pvcs *pre- and post-repeat PCR-RFLP results would reduce the total number of distinct genotypes to 55 with four isolates sharing the same genotype (highest genotype frequency = 0.054), and to 49 with seven isolates sharing the same genotype (highest genotype frequency = 0.095), respectively.

## Discussion

Populations of *P. vivax*, like those of other *Plasmodium *species, comprise genetically distinct lines that exhibit diversity in a number of factors of epidemiological, biological and clinical relevance. For example, the proportion of hypnozoites and the timing and frequency of their activation, differ between temperate and tropical strains of this parasite [28]. Resistance to drugs is linked to genetic mutations of defined genes [29] and recently the *Pvcs *repeat type was found to be associated with transmissibility by defined species of anophelines [30]. The ability to differentiate genetically different parasite populations would enhance a wide spectrum of investigations of the biology and epidemiology of *P. vivax*.

In this article, the potential of two *P. vivax *genes, *Pvcs *and *Pvmsp1*, as genetic markers, were assessed and practical protocols for their use in genotyping parasites collected from the field were described. PCR-based protocols targeted four polymorphic regions from two genetic markers, one from *Pvcs *and three from *Pvmsp1*, and were further associated to RFLP analyses of two of these regions, the repeat region of *Pvcs *and the F2 fragment of *Pvmsp1*, that allow the division of parasite populations into an incremental number of genetically distinct sub-groups.

For *Pvmsp1*, three polymorphic regions were independently assessed. The fragments F1, located at variable block 2 and F3 located at variable block 10, displayed moderate allelic size variation, five and four types respectively. The frequency of the size variants, in particular those of F3, displayed a biased frequency distribution among the 100 Thai isolates considered in this study, thus undermining their usefulness as genetic markers. Sequencing was carried out for a subset of F1 and F3 fragments amplified from the Thai isolates. This revealed that 13 F1 and 10 F2 allelic variants could actually be distinguished. These results confirm the high degree of complex polymorphisms observed for *Pvmsp1 *block 2 and block 10 observed in 31 isolates collected in Thailand, Brazil, Oceania and India, where 19 and 13 distinct allelic types were observed for block 2 and block 10 respectively [27]. Thus it is likely that the F1 and F3 segments could be exploited as useful genetic markers through the development of type-specific oligonucleotides, that would then be used in a series of nested PCR reactions. The amplified large central F2 fragment, located between variable block 6 and 8, showed two size variants only. However, when combined to RFLP analyses, using two restriction endonucleases, the variants were subdivided into a total of 36 different allelic types. For *Pvcs*, populations were divided into 10 subgroups through size and repeat type determination, and into 23 subgroups when sequence variations in the pre- and post-repeat sequences were assessed by RFLP.

The conclusion of this work is that the polymorphic repeat region of *Pvcs *and that of the *Pvmsp1 *F2 region can be considered suitable genetic markers of *P. vivax *populations, alone or in combination. This is supported by the fact that practical PCR-RFLP genotyping protocols revealed the presence of numerous distinct allelic variants in a sample of 100 isolates, among which they were randomly distributed without evidence of linkage disequilibrium between the two loci.

Interesting epidemiological indications could be derived from genotyping of the *P. vivax *isolates. Malaria is considered to be hypoendemic in Thailand where transmission, confined to regions bordering Cambodia, Lao PDR and Myanmar, rarely exceeds a few infective bites per person per year. A study of *P. falciparum *diversity, based on three genetic markers, in a refugee camp located in Thailand close to the Myanmar border, revealed levels of diversity and MOI (1.6, with mixed genotype infections observed in 60 % of the isolates) that were higher than expected from the low effective inoculation rates [31]. A recent study of *P. vivax *parasites collected in the same region and based on two genetic markers, showed that populations of this parasite species were also highly diverse and mixed genotype infections were observed in 35 % of the isolates [32]. The results of the present study indicated that 26 % of the isolates were of mixed genotype, with an overall MOI for all 100 isolates of 1.29. It should be stated that the three studies are not strictly comparable since the number and type of genetic markers, the genotyping protocols and the collection strategies that were employed differed. Furthermore, in the current study the origin of the *P. vivax *isolates was not confined to a single area, since the patients from whom blood was collected acquired their infection in diverse endemic areas of Thailand. Nonetheless, these studies indicated that the two biologically distinct parasite species have similar population characteristics. The production of infectious gametocytes early during the primary infection in *P. vivax *versus their late appearance, following the acute phase, in *P. falciparum*, and the existence of hypnozoites solely in *P. vivax*, might not be sufficient to explain the maintenance of diversity in areas of low transmission. Substantially prolonged duration of the infections, probably as a result of low level drug resistance leading to parasitological (though not clinical) failures, as suggested for *P. falciparum *[31], and/or an underestimation of the transmission intensity, might underlie the high genetic diversity and MOI observed for the two parasite species. These two non-mutually exclusive scenarios are consistent with the detection of a high level of mixed species infections observed in Thailand [33-36].

It is hoped that the methodologies presented here can be adopted as standard protocols for the genotyping of *P. vivax *parasites, not only for *in vivo *drug efficacy trials, but also in field-based investigations aimed at elucidating the biology, pathology and epidemiology of this important parasite species.

## Conclusion

These results indicate that the genotyping protocols presented can be useful in the assessment of *in vivo *drug efficacy clinical trials conducted in endemic areas and for epidemiological studies of *P. vivax *infections.

## List of Abbreviations used

*Pvcs*-Circumsporozoite surface protein gene of *Plasmodium vivax*

*Pvmsp1*-Merozoite surface protein 1 gene of *Plasmodium vivax*

*Pvmsp3α*-Merozoite surface protein 3 alpha gene of *Plasmodium vivax*PCR-Polymerase Chain Reaction

RFLP-Restriction Fragment Length Polymorphism

## Authors' contributions

GS, NJW and SP designed the study. GS and SP were responsible for the day-to-day supervision of the work. ACG, FL and LR and MI collaborated to obtain and analyze DNA sequences. SL and SP were responsible for patient recruitment and clinical management. MI developed the protocols with the help of GS, and carried out the vast majority of the laboratory work. GS and MI analyzed the data and composed the manuscript. All authors read and approved the final manuscript.
